# Correction: Development and extensive sequencing of a broadly-consented Genome in a Bottle matched tumor-normal pair

**DOI:** 10.1038/s41597-025-05752-9

**Published:** 2025-08-08

**Authors:** Jennifer H. McDaniel, Vaidehi Patel, Nathan D. Olson, Hua-Jun He, Zhiyong He, Kenneth D. Cole, Alexander A. Gooden, Anthony Schmitt, Kristin Sikkink, Fritz J. Sedlazeck, Harsha Doddapaneni, Shalini N. Jhangiani, Donna M. Muzny, Marie-Claude Gingras, Heer Mehta, Sairam Behera, Luis F. Paulin, Alex R. Hastie, Hung-Chun Yu, Victor Weigman, Alison Rojas, Katie Kennedy, Jamie Remington, Isai Salas-González, Mitch Sudkamp, Kelly Wiseman, Bryan R. Lajoie, Shawn Levy, Miten Jain, Stuart Akeson, Giuseppe Narzisi, Zoe Steinsnyder, Catherine Reeves, Jennifer Shelton, Sarah B. Kingan, Christine Lambert, Primo Baybayan, Aaron M. Wenger, Ian J. McLaughlin, Aaron Adamson, Christopher Kingsley, Melanie Wescott, Young Kim, Benedict Paten, Jimin Park, Ivo Violich, Karen H. Miga, Joshua Gardner, Brandy McNulty, Gail L. Rosen, Rajiv McCoy, Francesco Brundu, Erfan Sayyari, Konrad Scheffler, Sean Truong, Severine Catreux, Lesley Chapman Hannah, Doron Lipson, Hila Benjamin, Nika Iremadze, Ilya Soifer, Gat Krieger, Stephen Eacker, Mary Wood, Erin Cross, Greg Husar, Stephen Gross, Michael Vernich, Mikhail Kolmogorov, Tanveer Ahmad, Ayse G. Keskus, Asher Bryant, Francoise Thibaud-Nissen, Jonathan Trow, Jacqueline Proszynski, Jeremy Wain Hirschberg, Krista Ryon, Christopher E. Mason, Mital S. Bhakta, J. Zachary Sanborn, Elizabeth M. Munding, Justin Wagner, Chunlin Xiao, Andrew S. Liss, Justin M. Zook

**Affiliations:** 1 Material Measurement Laboratory, National Institute of Standards and Technology, 100 Bureau Dr., Gaithersburg, MD 20899 USA; 2 Arima Genomics, Inc., 6354 Corte Del Abeto Suite B, Carlsbad, CA 92011 USA; 3 Department of Molecular and Human Genetics, Baylor College of Medicine, Houston, TX USA; 4 Department of Computer Science, Rice University, 6100 Main Street, Houston, TX USA; 5 Human Genome Sequencing Center, Baylor College of Medicine, One Baylor Plaza, Houston, Texas 77030 USA; 6 Bionano Genomics, San Diego, USA; 7 BioSkryb Genomics, 2810 Meridian Pkwy, ste110, Durham, NC 27713 USA; 8 Applications Development Laboratory, Element Biosciences, 10055 Barnes Canyon Rd, San Diego, CA 92121 USA; 9 Genome Technology Laboratory, Northeastern University, 360 Huntington Ave, Boston, MA 02115 USA; 10 New York Genome Center, New York, NY 10013 USA; 11 PacBio, 1305 O’Brien Drive, Menlo Park, CA 94025 USA; 12 UC Santa Cruz Genomics Institute, University of California, Santa Cruz, CA USA; 13 Ecological and Evolutionary Signal-processing and Informatics (EESI) Lab, College of Engineering, Drexel University, Philadelphia, PA USA; 14 Department of Biology, Johns Hopkins University, 3400 N. Charles St., Baltimore, MD 21218 USA; 15 Illumina, Inc., DRAGEN, 5200 Illumina Way, San Diego, CA 92122 USA; 16 National Cancer Institute, Division of Cancer Epidemiology and Genetics, Clinical Genetics Branch, National Institutes of Health, 9609 Medical Center Dr, Rockville, MD 20850 USA; 17 Ultima Genomics Inc., 4425 Technology Dr, Fremont, CA 94538 USA; 18 Phase Genomics, Inc, Seattle, WA USA; 19 KROMATID, 1880 Industrial Circle, Suite A, Longmont, Colorado, 80501 USA; 20 Cancer Data Science Laboratory, National Cancer Institute, National Institutes of Health, 9000 Rockville Pike, Bethesda, MD 20892 USA; 21 National Center for Biotechnology Information, National Library of Medicine, National Institutes of Health, 45 Center Drive, Bethesda, MD 20894 USA; 22 Weill Cornell Medicine, Physiology and Biophysics and WorldQuant Initiative, 1300 York Avenue, New York, NY 10021 USA; 23 Dovetail Genomics, part of Cantata Bio, 100 Enterprise Way, Suite A101, Scotts Valley, CA 95066 USA; 24 Department of Surgery, Massachusetts General Hospital and Harvard Medical School, Boston, MA 02114 USA

**Keywords:** Genome informatics, Cancer genomics

Correction to: *Scientific Data* 10.1038/s41597-025-05438-2, published online 16 July 2025

In this article the following sentence was omitted from the Usage Notes, ‘The data and alignments are publicly available on the GIAB FTP site hosted by the NIH-NCBI https://ftp.ncbi.nlm.nih.gov/ReferenceSamples/giab/data_somatic/HG008/’. The original article has been corrected.

